# Establishing a Percutaneous Infection Model Using Zebrafish and a Salmon Pathogen

**DOI:** 10.3390/biology10020166

**Published:** 2021-02-22

**Authors:** Hajime Nakatani, Katsutoshi Hori

**Affiliations:** Graduate School of Engineering, Nagoya University, Furo-cho, Chikusa, Nagoya 464-8603, Japan; nakatanih@chembio.nagoya-u.ac.jp

**Keywords:** bacterial flora, fish skin, fish pathogen, *Yersinia ruckeri*

## Abstract

**Simple Summary:**

The epidermis and mucus layer of fish act as barriers that protect them against waterborne pathogens, and provide niches for symbiotic microorganisms that benefit the host’s health. However, our understanding of the relationship between fish skin bacterial flora and fish pathogen infection is limited. In order to elucidate this relationship, an experimental model for infection through fish skin is necessary. Such a model must also pose a low biohazard risk in a laboratory setting. We established a percutaneous infection model using zebrafish (*Danio rerio*), a typical fish experimental model, and *Yersinia ruckeri*, a salmon pathogen. Our experimental data indicate that *Y. ruckeri* colonizes niches on the skin surface generated by transient changes in the skin microflora caused by stress, dominates the skin bacterial flora, occupies the surface of the fish skin, invades the fish body through injury, and finally, causes fatal enteric redmouth disease. This percutaneous infection model can be used to study the interaction between fish skin bacterial flora and fish pathogens in water, or the relationship between pathogens and the host’s skin immune system.

**Abstract:**

To uncover the relationship between skin bacterial flora and pathogen infection, we developed a percutaneous infection model using zebrafish and *Yersinia ruckeri,* a pathogen causing enteric redmouth disease in salmon and in trout. Pathogen challenge, either alone or together with pricking by a small needle, did not cause infection of the fish. However, cold stress given by water temperature shift from the optimum 28 °C for zebrafish to 20 °C caused fatal infection of injured fish following pathogen challenge. We investigated the effects of cold stress, injury, and pathogen challenge, alone and in combination, on fish skin bacterial flora using 16S rDNA metagenomics. We found that cold stress drastically altered the skin bacterial flora, which was dominated by *Y. ruckeri* on infected fish. In addition, fish whose intrinsic skin bacterial flora was disrupted by antibiotics had their skin occupied by *Y. ruckeri* following a challenge with this pathogen, although the fish survived without injury to create a route for invasion into the fish body. Our results suggest that the intrinsic skin bacterial flora of fish protects them from pathogen colonization, and that its disruption by stress allows pathogens to colonize and dominate their skin.

## 1. Introduction

As fish live in aquatic environments and are constantly exposed to microorganisms, their skin is a critical first line of defense against pathogen infection. The epidermis and the mucus layers of fish skin act as barriers that protect fish from pathogens in water. Moreover, they also provide niches for symbiotic microorganisms, which have positive effects on the host’s health [1,2,3]. The symbiotic microorganisms on fish skin are considered to have protective effects against pathogens. Basic studies of fish skin microflora have demonstrated that it is altered by handling in fish farms and by changes in environmental conditions, including pH, salt concentration, and temperature [4,5,6,7,8]. However, our understanding of the relationship between the fish skin bacterial flora and fish pathogen infection is lacking [9]. Uncovering this relationship could facilitate infection control utilizing the fish skin bacterial flora. 

A laboratory infection model is necessary to obtain reproducible and reliable experimental data from analyses of biological samples taken from individuals in different growth conditions. Infection experiments on fish should be conducted under controlled environments. However, it is quite difficult to control environmental parameters in the field affected by the climate and by seasonal weather. In addition, the use of fish pathogens in open environments such as fish farms is legally regulated because of pandemic potential. Experiments with pathogens must therefore be performed in special facilities to limit biohazardous risks. However, it is difficult to collect many individuals of common farmed fish with identical physiological conditions, such as growth stage, size, and genetic background, for experiments in laboratories. 

The purpose of this study is to establish a percutaneous infection model using zebrafish (*Danio rerio*) and *Yersinia ruckeri*. *D. rerio* is a commonly used experimental model fish that is easy to grow in a small lab space, and is suitable for studies concerning infectious disease and the role of microbiota in infectious disease [10,11]. Moreover, genetic, physiological, and immunological information about zebrafish is abundant and useful for understanding host response to pathogens during infection. *Y. ruckeri* is known to cause enteric redmouth disease (ERM) in fish, mainly among salmon and trout [12]. The incidence of *Y. ruckeri* infection among farmed fish is rising around the world. Although gills are the main infection route for *Y. ruckeri* [12], this bacterium was recently found on the skin of infected rainbow trout [13]. We searched experimental manipulations that reproducibly caused *Y. ruckeri* infection of zebrafish and performed 16S rDNA metagenomics of fish skin microflora to confirm the percutaneous infection accompanied by changes in the skin bacterial flora.

## 2. Materials and Methods

### 2.1. Maintenance and Handling of Zebrafish

Adult zebrafish used for the infection experiments (about 1 year old, 3–4 cm, 2:3 of male: female ratio, 300–400 fish) were acquired from the lab stock of Mie University. They were kept in a CROSS MINI (NWC-341; NISSO, Osaka, Japan) aquarium system for more than 2 weeks before being subjected to infection experiments. The fish (40 fish/6.8 L tank) were fed TetraMin Super 17653 (Spectrum Brands Japan, Kanagawa, Japan) every 12 h using a Tetra Auto Feeder AF-3 (Spectrum Brands Japan). A 12:12-h light-dark cycle was established using a Tetra LED Mini Light (Spectrum Brands Japan) for a tank and a digital timer PT70DW (REVEX, Saitama, Japan). Breeding water (6.8 of pH, 5° dH of carbonate hardness (KH), 0° dH of total hardness (GH), more than 7.0 mg/L of dissolve oxygen (DO), 18 L/tank) was maintained at 28 °C by using Safe Cover Heat Navi SH80 (GEX, Osaka, Japan).

When treating zebrafish with antibiotics, ampicillin (final concentration: 100 μg/mL), kanamycin (final concentration: 50 μg/mL), and amphotericin B (final concentration: 100 μg/mL) were added to 200 mL of sterile breeding water in a 500 mL flask, and five zebrafish were placed in it for 24 h at 20 °C. The breeding water was replaced with new water before pathogen challenge at 20 °C. To euthanize zebrafish, fish were placed in a tricaine (3-amino benzoic acid ethyl ester: TCI, Tokyo Japan) solution (600 mg/L) and allowed to swim until they stopped moving.

### 2.2. Bacterial Strains

*Y. ruckeri* NVH 3758 was isolated from rainbow trout with ERM in Norway, and kindly provided to us by Dr. Linke. For infection experiments, the strain was cultured in LB medium (Miller) at 28 °C for 24 h with shaking at 115 rpm. *Aeromonas hydrophila* NRIA14 and *Vibrio ordalii* NRIA90 were provided from the Japan Fisheries Research and Education Agency. *Aeromonas caviae* JCM1043 was purchased from the Japan Collection of Microorganisms, RIKEN BioResource Center.

*Y. ruckeri* transformation was performed by conjugal transfer from *Escherichia coli* SM10 (λpir [14] harboring pLBT::mini-Tn*10*:*lac*:*kan* transposon vector [15]. *Y. ruckeri* transformants were isolated as previously described [16].

To isolate bacterial strains from the intrinsic skin microflora of zebrafish, euthanatized fish were laid on an aluminum foil disinfected with 70% ethanol, and the mucus on the fish epidermis was scraped with cotton swabs. The swabs were immersed in 1 mL Ultrapure deionized water. The collected mucus was resuspended using a vortex mixer. An aliquot (200 μL) of either the original suspension or their dilutions were spread on a nutrient agar medium, enriched Cytophaga agar medium, modified Zobell 2216E [17] agar medium (0.8% NaCl), or tryptone soya broth agar medium, and incubated at either 28 °C or 20 °C for 2 d to grow bacterial colonies.

### 2.3. Detection of Bacteria

PCR detection of *Y. ruckeri* was performed using 16S ribosomal DNA (rDNA) V1–V2 region specific primers or *Y. ruckeri glnA* (glutamine synthetase) specific primers [18]. Primer sequences are summarized in Table S1. Amplification of target genes was verified by agarose gel electrophoresis and with an Agilent 2100 Bioanalyzer (Agilent Technologies, Santa Clara, CA, USA).

*Y. ruckeri* adhering to fish skin surface was observed through in vivo imaging of *lacZ*-expressing *Y. ruckeri*. For the imaging, zebrafish were euthanized, as described above, and fixed with 4% paraformaldehyde in phosphate-buffered saline (PBS) (pH 7.2) at 4 °C for 30 min. After washing the fish once with distilled water, the fish were placed in PBS containing 0.1% Triton-X 100 for 1 h. The fish were subsequently transferred to an X-gal staining solution (30 mM K_3_[Fe(CN)_6_], 30 mM K_4_[Fe(CN)_6_], 2 mM MgCl_2_, 1 mg/mL X-gal, 0.05% Tween-20 in PBS at pH 7.2) and maintained for 5 h at 30 °C. Fixed fish were observed under a fluorescence stereomicroscope (Axio Zoom V16, Carl Zeiss, Oberkochen, Germany).

### 2.4. Growth Inhibition Tests

To determine the growth inhibition activities of fish pathogens, *A. caviae* JCM1043, *A. hydrophila* NRIA14, *V. ordalii* NRIA90, *Y. ruckeri* NVH 3758, and isolated strains from the fish skin above were pre-cultured in a liquid medium overnight. The cross-streak method was performed, as described previously [19]. In short, one of the isolated strains was streaked vertically on the solid medium that was used for its isolation, and the fish pathogens were streaked horizontally to cross the line of the isolated strain on the same agar medium. The plates were incubated thereafter at 28 °C or 20 °C for a few days.

### 2.5. Infection Experiments

Sterile Instant Ocean Sea Salt (NAPQO, Concord, OH) solution was added to sterile water to prepare sterile breeding water, which contains 3 g/L seawater salt (10% the salt content of seawater). Three mL of a tricaine (3-amino benzoic acid ethyl ester: TCI, Tokyo Japan) solution (2 mg tricaine dissolved in 1 mL of 0.021 M Tris) was added to 100 mL of sterile breeding water to make an anesthetic solution. The fish were allowed to swim in the anesthetic solution for several minutes. Anesthetized fish were laid on an aluminum foil disinfected with 70% ethanol. Fish were injured by stinging at a dorsal ridge behind the dorsal fin with an injection needle (NN-2613S, 26 gauge, 13 mm; Terumo, Tokyo, Japan). The five injured fish were transferred into a 500 mL flask with 200 mL of the sterile breeding water, and maintained until they recovered from anesthesia. They were kept for 24 h in the flask with aeration at either 20 °C or 28 °C. As a control group, fish without injury and pathogen challenge (injury−, *Y. ruckeri*−) were also simultaneously maintained in a flask under the above conditions. After 24 h, a bacterial suspension of *Y. ruckeri* at an OD_600_ (optical density at 600 nm) of 1.0 in Ultrapure deionized water was added to the flasks containing a fish group with pathogen challenge so that the OD_600_ in breeding water was reduced to 0.01. After exposure to the pathogen for 6 h, the fish were transferred to fresh sterile breeding water in a new flask and maintained up to seven days at either 20 °C or 28 °C for the observation of an infected state. Feeding was discontinued for the duration of the infection experiment. Of five to ten fish, the numbers of dead and live fish were counted in each experimental set to calculate the survival rate and Kaplan-Meier curves were figured by executing R script on Rstudio (https://rstudio.com/, accessed on 20 September 2019). The log rank tests were also executed on Rstudio. The infection experiments were repeated three times in each experimental set. 

Infection by *Y. ruckeri* was determined by either the observation of symptoms specific to ERM or by death of the fish. Fish were examined for disease symptoms every 12 h. Symptoms included slow swimming caused by debilitation as well as redness or petechiae at the lower jaw or the base of the fin [12]. *Y. ruckeri* infection was diagnosed when these symptoms were observed. Fish with ERM symptoms and dead fish were captured immediately from the flasks. At the same time, the same number of fish in the control group were captured. All fish that survived for one week after pathogen challenge were also captured for further experiments. In addition, the breeding water was collected at the end of the infection experiment and filtered with suction through a membrane filter (mixed cellulose, 0.45 μm pore size, 47 mm diameter; ADVANTEC, Tokyo, Japan).

### 2.6. Next-Generation Sequencing of 16S rDNA Amplicon Libraries

A total of 45 zebrafish from ten experimental conditions (4–5 fish par each condition) were euthanized, as described above, rinsing off breeding water around the fish skin. Skin bacterial floras were collected by peeling off the skin of the fish, and skin samples containing the floras were transferred to sterile 1.5 mL tubes and stored at −30 °C. Bacteria in breeding water from seven experimental conditions were collected by filtration with 0.45 µm filter. A NucleoSpin Tissue Kit (Takara, Otsu, Japan) was used to extract and purify genomic DNA from skin samples containing surface microorganisms. According to the protocol for recovering bacterial genomic DNA from difficult-to-lyse bacteria, such as Gram-positive bacteria, the sample was pretreated with a pre-lysis solution (20 mg/mL lysozyme; 127-06724; Wako Pure Chemical Industries, 20 mM Tris-HCl, 2 mM EDTA, 1% Triton X-100) at 37 °C for 1 h. Bacterial 16S rDNA amplicon libraries were prepared from the extracted genomic DNA for DNA sequencing using iSeq 100 (Illumina, San Diego, CA, USA) according to the manual (16S Metagenomic Sequencing Library Preparation; 15044223 Rev. A; Illumina) with slight modifications. Ex Taq (Takara Bio, Kusatsu, Shiga, Japan) was used to amplify either the 16S rDNA V4 region from the extracted genomic DNA. Primer sequences for specific amplification of the V4 region were selected from sequences introduced in the Earth Microbiome Project (Table S1). Overhang sequences for adaptor extension were added to the region-specific primer sequence. Primer sequences are summarized in supplemental Table S1. To eliminate the contaminated DNA in reagents, 12.5 µg/mL of 5-methoxypsoralen was added to a PCR reagent mix, and the contaminated DNAs were cross-linked by exposing 365 nm UV for 10 min. Reagent samples without DNA were subjected to PCR for negative controls. The temperatures for denaturation, annealing, and extension reaction were set at 95 °C, 50.7 °C, and 72 °C, respectively. After 25 cycles of PCR, 11 of 52 samples were randomly selected, and the sizes of the amplification products (about 250–270 bp without primer sequences) were confirmed using an Agilent 2100 Bioanalyzer. The amplicons were purified using Agencourt AMPure XP beads (A63881; Beckman Coulter, Brea, CA, USA). In eight cycles of PCR using the Nextera XT Index Kit (Illumina) and 2× KAPA HiFi HotStart ReadyMix (Roche Diagnostics, Basel, Switzerland), the adapter and index sequences for Illumina sequencing analysis were added to the amplicons. The temperature conditions for this second PCR were as follow: 95 °C for denaturation, 55 °C for annealing, and 72 °C for extension reaction. The adapter-added amplicon libraries were again purified using AMPure XP beads. A part of the libraries was analyzed by the Bioanalyzer to confirm the library size. The library concentration was measured using the Qubit 4 and its dsDNA BR Assay Kit (Thermo Fisher Scientific, Waltham, MA, USA). The library concentration was finally adjusted to 50 pM for analysis by the iSeq100 Sequencing System (1000000036024 v03 JPN; Illumina). Twenty µL of PhiX Control v3 (Illumina) was added to 100 µL of the library (about 16% of the library volume) to enhance sequence data quality.

### 2.7. In Silico Analysis Based on 16S rDNA Amplicon Sequencing

CLC Genomic Workbench (Qiagen Japan, Tokyo, Japan) with the Microbial genomic module was used for bacterial flora analysis and statistical analysis based on 16S rDNA sequences obtained by the iSeq100. The analyses were performed using sense reads (about 150 bp) of the pair-end fastq, because the merged sequences were not adequately obtained. Sequence data were imported into the software, and the length of the reads was trimmed using the ‘Trimming Sequence’ tool. As parameters, the length of the sequence after trimming was set to a minimum of 140 bp and a maximum of 160 bp, the base calling error probability limit was set to 0.05, and the maximum number of indeterminate bases was set to 1. Reads that matched the zebrafish reference genome (mitochondrial DNA etc.) were removed by the ‘Clean Host DNA’ tool. In order to classify reads according to experimental conditions, metadata files that included parameters for sample ID, water temperature, the presence or absence of injury, presence or absence of pathogen challenge, and live versus dead were created. 

Reference-based operational taxonomic unit (OTU) clustering was performed utilizing the ‘OTU Clustering’ tool. The function of the elimination of chimera reads was executed during ‘OTU Clustering’, and the reads from 16S rDNA (1500–20,000 reads) were classified into OTUs. The Greengenes database (http://greengenes.lbl.gov, accessed on 14 December 2018) was used as the reference database using a similarity threshold of 97%. The abundance tables including OTU data were exported as a Microsoft Excel spreadsheet (Data S1), and the total number of reads, the number of OTUs, and the number of OTUs duplicated between samples were calculated. In order to identify bacterial species from the read sequences, we searched for sequences similar to the read sequences in the NCBI 16s rDNA database using BLAST.

Analyses of β-diversity among experimental groups were performed as follows. An abundance table was created from a set of the sequence data to be compared, and the reads classified into OTUs were aligned using the ‘Align OTUs with MUSCLE’ tool. A phylogenetic tree was generated based on the alignment of reads using the ‘Create tree’ tool and the neighborhood linkage method. The K80 model [20] was used to measure distances based on nucleic acid sequences’ alignment. The calculated weighted Unifrac distance between each sample [21] was obtained from the phylogenetic tree generated using the ‘Beta diversity’ tool, and three-dimensional principal coordinate (3D PCoA) analysis was performed. 

PERMANOVA (PER mutational Multivariate Analysis Of Variance) analysis for determining the intergroup variability and the statistical analysis for intergroup difference was performed. The weighted Unifrac distance was used for distance metrics. The permutation (number of permutations) was set to 99,999. The statistical value was calculated by the method of Anderson et al. [22].

Heat maps were generated using the ‘Create Heat Map for Abundance Table’ tool based on the abundance of OTUs in the respective samples. An abundance table was generated from the sequence data of the samples to be compared as described above. The Euclidean distance was used for distance metrics between two samples, and the perfect connection method was selected as a clustering method. 

Alpha-diversity indices were calculated using specific tools in CLC Genomic Workbench [23,24,25]. To generate rarefaction curves, the range of the sub-sampling depth was set from 1 to 1500 reads, the number of different depths for sampling was set to 50, and the number of replicates for each depth was set to 100. The results of rarefaction curves were exported as Microsoft Excel spreadsheets (Data S2) and the results at the maximum sub-sampling depth (1500 reads) were presented as box plots.

## 3. Results

### 3.1. Conditions That Permit Percutaneous Infection of Zebrafish by Y. ruckeri

**Figure 1:**
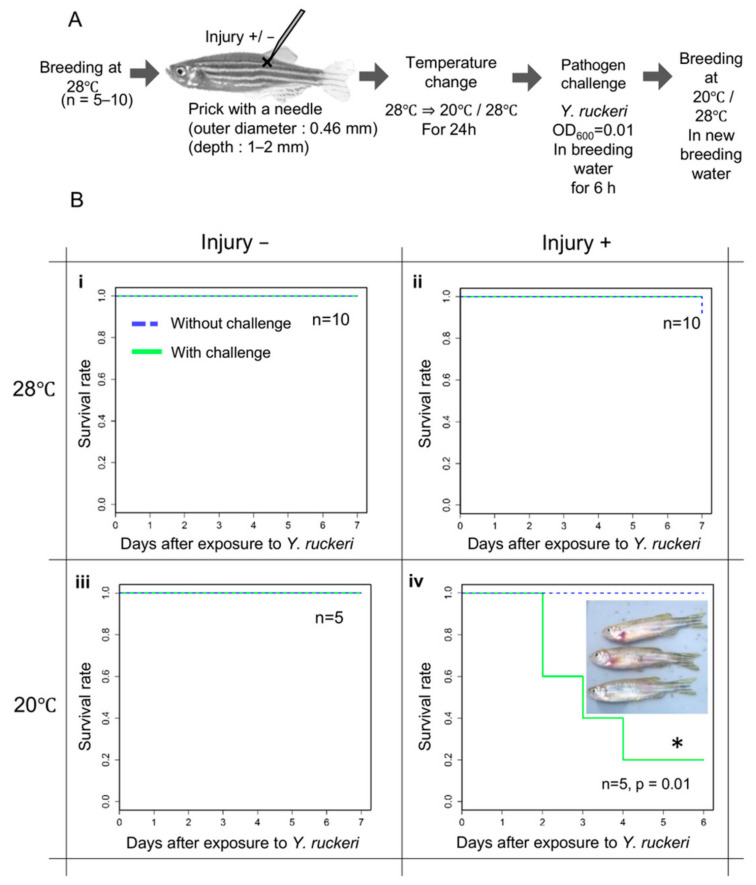
Procedure and results of infection experiments. (**A**) Schematic procedure of infection experiments. (**B**) The survival rate of zebrafish in infection experiments. Surviving fish under each experimental condition were counted daily for seven days after being challenged with *Y. ruckeri*. The survival rate of zebrafish subjected to pathogen challenge (green line) was compared with that of zebrafish that were not challenged (blue dotted line). Inset image: dead fish previously challenged with *Y. ruckeri*. *; Significant difference against a control group by log rank test.

As the virulence of *Y. ruckeri* toward zebrafish is unknown, we first determined the conditions for effective infection of zebrafish by *Y. ruckeri* (Figure 1A). To construct a percutaneous infection model, we challenged zebrafish with the pathogen by immersing the fish in breeding water containing *Y. ruckeri* for several hours. After immersion, the fish were maintained in breeding water without the pathogen. Pathogen challenge alone did not cause the infection of zebrafish with *Y. ruckeri*, and all of the fish survived without showing symptoms of ERM (Figure 1B(i)).

The fish were subsequently injured by pricking with a needle at the epidermis near the dorsal fin to provide an invasion route for the pathogen from the body surface. Injured fish were immersed in breeding water containing the pathogen. However, despite the injury, no fish died (Figure 1B(ii)). 

Next, 24 h before pathogen challenge, we changed the breeding water temperature from 28 °C, the optimum for zebrafish, to 20 °C in order to apply cold stress to the fish. Without injury, cold stress was not conducive to pathogen infection (Figure 1B(iii)). Incidentally, when the breeding temperature was further lowered to 15 °C, all the fish died regardless of the presence or absence of pathogen. However, injured zebrafish kept under cold stress at 20 °C were infected after being challenged with *Y. ruckeri* at an OD_600_ of 0.01 for 6 h. Under these conditions, most fish with injury died within seven days of pathogen challenge at 20 °C (Figure 1B(iv)). The dead fish all showed hemorrhages around the gills and symptoms of ERM. 

**Figure 2:**
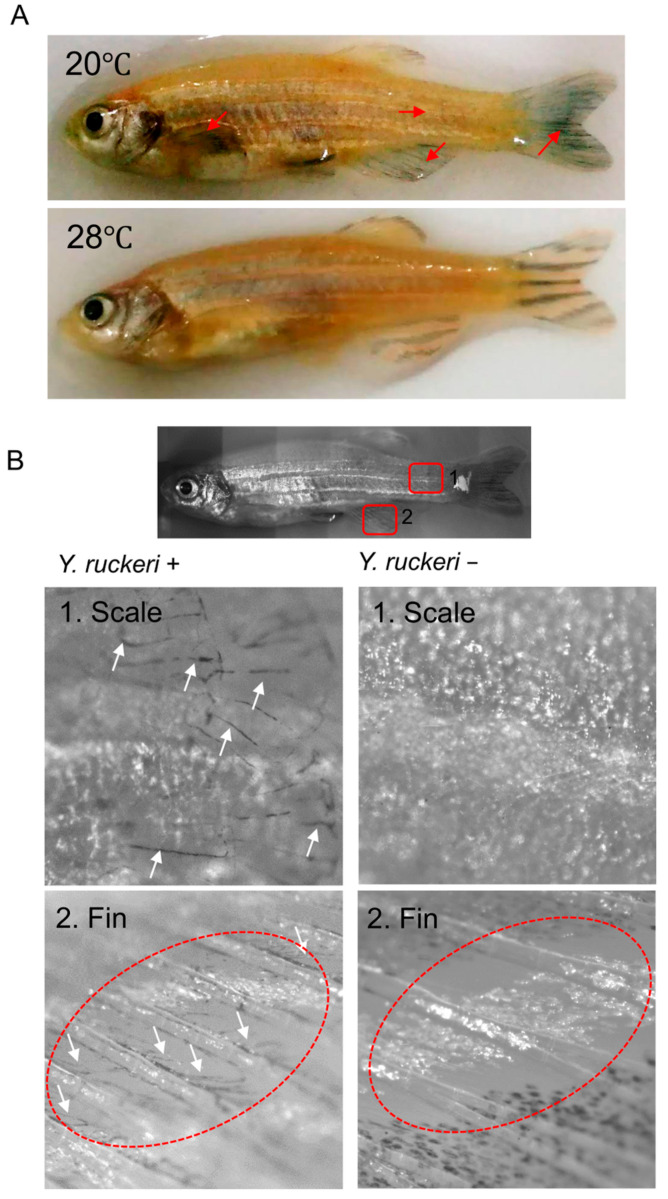
In vivo imaging of the *Yersinia ruckeri* on the fish skin. (**A**) Whole views of zebrafish 48 h after pathogen challenge at 28 °C and 20 °C. Red arrows represent the positive areas showing X-gal staining of *Y. ruckeri* expressing *lacZ*. (**B**) Detailed observation of fish with (*Y. ruckeri* +) and without (*Y. ruckeri*−) pathogen challenge at 20 °C under a microscope. Microscopic images of scales around a tail fin (1) and an anal fin (2) are shown. *Y. ruckeri* cells stained with X-gal were seen as black dots along with uneven patterns of the surface (white arrows).

We verified the presence of *Y. ruckeri* on the skin of both dead and live fish. DNA samples extracted from fish skin and skinborne microorganisms were subject to PCR using a pathogen-specific primer set. We thereby detected *Y. ruckeri* on the skin of dead fish (Figure S1). *Y. ruckeri* cells were also visualized in vivo by overexpressing *lacZ.* Colonization by the pathogen was verified 48 h after pathogen challenge on the surface of fins and scales in zebrafish that were still alive under infectious conditions, while *Y. ruckeri* cells could not be detected in fish under survival conditions at 28 °C (Figure 2). 

### 3.2. Effect of Y. ruckeri Colonization on Zebrafish Skin Microflora

In order to determine how the skin microflora was affected by *Y. ruckeri* colonization, we analyzed the composition of the skin bacterial floras of zebrafish in our infection experiments. Comprehensive bacteria-derived 16S rDNA sequencing data were obtained from each fish’s skin sample in our infection experiments. We obtained a total of 401,858 reads from the collected skin samples. These sequence data were collated with the reference database and classified into OTUs. Sample reads were thereby classified into 632 OTUs. 

**Figure 3:**
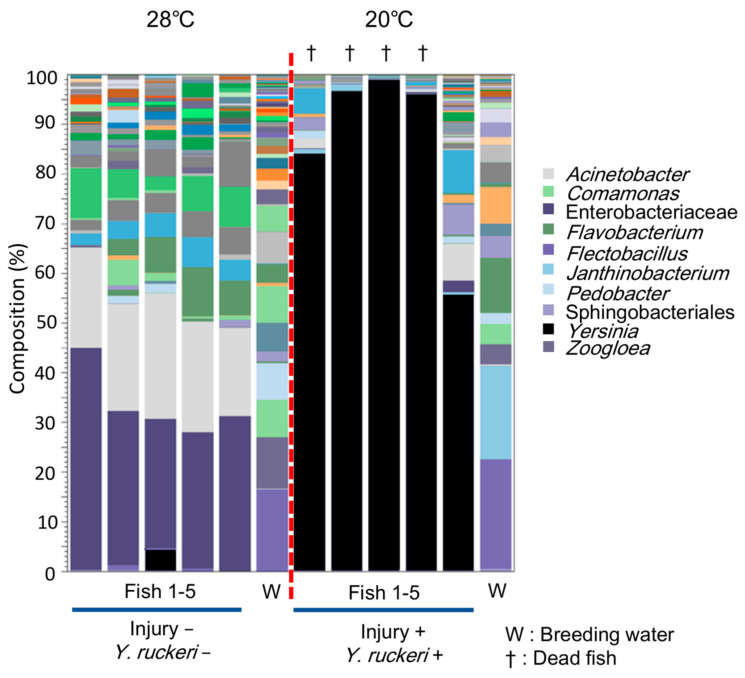
Composition of skin bacterial floras from zebrafish under normal breeding conditions and under infectious conditions. The stacked bar chart shows the composition operational taxonomic units (OTUs, top 50) in the fish skin bacterial flora above the genus level. The bars to the left and right of the red dot line represent data collected under normal breeding conditions and infectious conditions, respectively.

Figure 3 compares the composition of bacterial groups in skin microfloras obtained from infected fish described above against those from fish in a normal breeding condition (28 °C) without any experimental manipulation (injury−; *Y. ruckeri*−;). Our results indicate that the skin of infected fish was occupied by bacteria belonging to genus *Yersinia*; the occupancy rate rose to more than 80% from several dead fish samples. Even in the only surviving individual under infectious conditions (injury+; *Y. ruckeri*+; 20 °C), *Yersinia* spp. was dominant (>55%) among the skin bacterial flora. Thus, *Y. ruckeri* occupied the skin bacterial flora of zebrafish prior to death. In contrast, the genus *Yersinia* did not predominate on zebrafish in the normal breeding condition. These data suggest that *Y. ruckeri* infects zebrafish percutaneously by adhering to and colonizing the fish skin under infectious conditions.

### 3.3. Effects of Experimental Manipulations on Fish Skin Bacterial Flora

**Figure 4:**
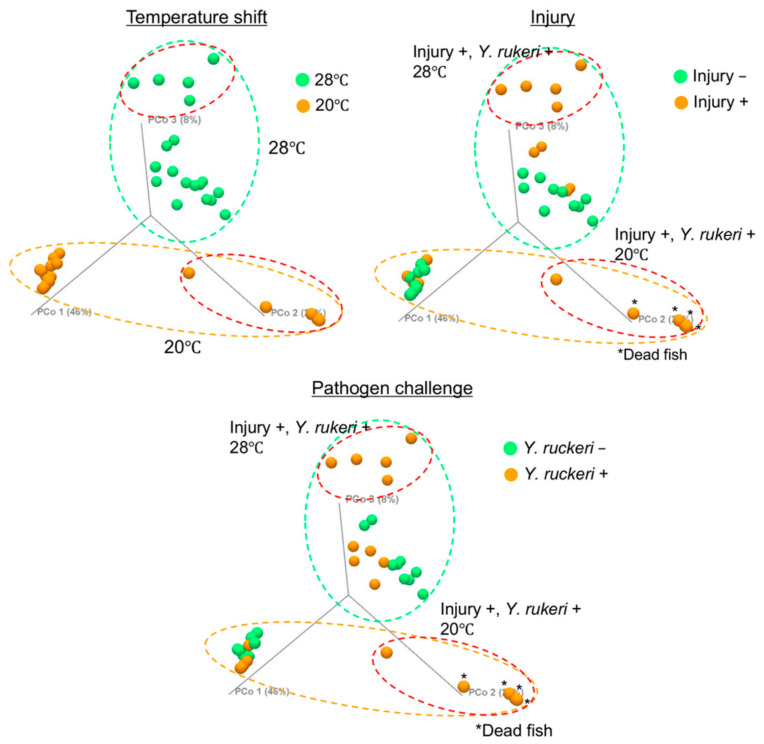
β-diversity of the skin bacterial floras on zebrafish subjected to experimental manipulation. Data are shown in a 3D-PCoA plot. Green dotted circles, orange dotted circles, and red dotted circles represent the fish grouped by the test temperature 28 °C, the test temperature 20 °C, and experimental manipulation (injury and pathogen challenge), respectively.

We next investigated which experimental manipulation we subjected fish to (i.e., cold stress (temperature shift), injury, or pathogen challenge) affected fish skin bacterial flora to the greatest degree. To determine differences in the composition of bacterial floras in the experimental manipulation groups, we conducted β-diversity analysis of the bacterial floras using the weighted Unifrac distance [21] and visualized the differences by 3D-PCoA (Figure 4 and Figure S2, Table S2). According to 3D-PCoA, the change in bacterial flora composition was due mainly to the change in breeding water temperature. Applying pathogen challenge to injured fish affected the skin bacterial floras regardless of temperature tested. However, the observed changes in the composition of bacterial flora caused by either injury or pathogen challenge alone were small. 

**Figure 5:**
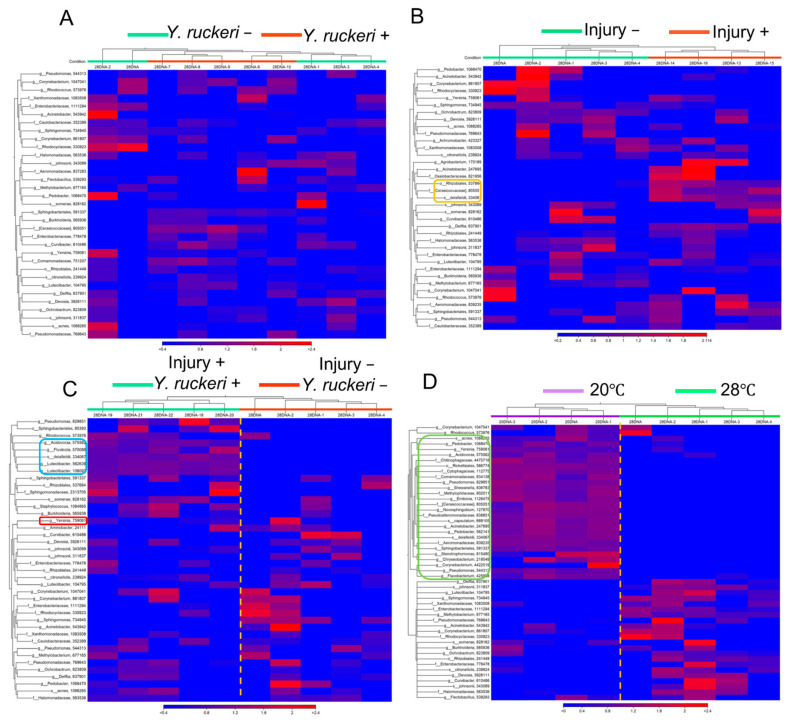
Heatmaps showing the relative abundances of OTUs (top 50) in skin bacterial floras. (**A**) Samples from fish with (injury−, *Y. ruckeri*+) or without pathogen challenge (injury−, *Y. ruckeri*−) at 28 °C without injury. (**B**) Samples from injured (injury+, *Y. ruckeri*−) and uninjured fish (injury−, *Y. ruckeri*−) at 28 °C without pathogen challenge. An orange frame shows OTUs that increased following injury. (**C**) Samples from fish subjected to double manipulations of injury and pathogen challenge (injury+, *Y. ruckeri*+) and fish that were not subject to these manipulations (injury−, *Y. ruckeri*−). Both groups of fish were maintained at 28 °C. A blue frame shows OTUs increased by the double manipulations. A red frame shows the genus *Yersinia*. (**D**) Samples from fish maintained at 28 °C and 20 °C that were not subjected to either injury or pathogen challenge. A green frame shows OTUs increased at 20 °C compared with 28 °C.

To determine the differential effects of injury and/or pathogen challenge or temperature shift on major taxonomic components of the skin bacterial flora, clustered heatmaps were constructed from the abundance of major OTUs (Top 50) collected from individual fish samples in the experiments. Comparing heatmaps from a fish subjected to pathogen challenge (injury−, *Y. ruckeri*+) with a fish that was not challenged (injury−, *Y. ruckeri−*) at 28 °C indicated that pathogen challenge has no detectable effects on the abundance of OTUs on uninjured fish in the absence of cold stress (Figure 5A). In the absence of both pathogen challenge and cold stress, pricking fish has small effects on the abundance of OTUs on fish, which is indicated by the comparison of heatmaps between two fish groups with injury (injury+, *Y. ruckeri−*) and without injury (injury−, *Y. ruckeri−*); however, the abundance of some OTUs, such as Rhizobiales, Cerasicoccaceae, and *Acidovorax delafieldii,* increased following injury (Figure 5B).

Since the effect of either pathogen challenge or injury alone on the main OTUs among the skin bacterial flora was unclear, we constructed a heatmap to compare the main OTUs between fish subjected to both injury and pathogen challenge (injury+, *Y. ruckeri*+) and fish that were not subjected to either of these manipulations (injury−, *Y. ruckeri*−) at 28 °C. Our heatmap shows that injury combined with pathogen challenge affected the abundance of the main OTUs but that the effect was not drastic (Figure 5C). The abundance of both genus *Luteolibacter* and genus *Acidovorax* increased but that of genus *Yersinia* did not follow the pathogen challenge on injured fish skin. In contrast to the effects of injury and/or pathogen challenge, a drastic transition in the major OTUs in the skin bacterial flora was caused by the shift in temperature from 28 °C to 20 °C even in the absence of injury or pathogen challenge (Figure 5D). The OTUs from major bacterial genera differed almost entirely in fish at these different temperatures.

**Figure 6:**
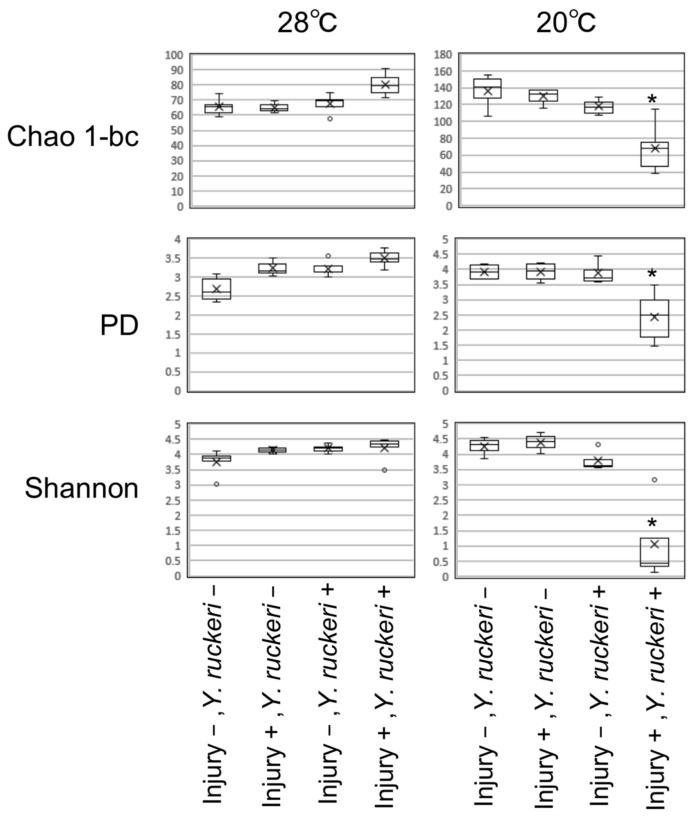
α-diversity of skin bacterial floras by injury and pathogen challenge at each temperature. The diversity indices, bias-corrected Cho1 estimator (Chao1-bc), phylogenetic diversity (PD), and Shannon diversity index (Shannon), were calculated by rarefaction analysis using 1500 reads selected randomly from each sample. Results from 3–5 individuals in each condition were represented by the box plot. *; *p* < 0.05 against a control group by Mann–Whitney U test.

The α-diversity indices, important indicators for the stability of microflora against perturbation [23,24,25], were analyzed about the skin bacterial floras of fish that underwent experimental manipulations (injury alone, pathogen challenge alone, or both injury and pathogen challenge) and were compared with those of control fish not subjected to either injury or pathogen challenge (injury−, *Y. ruckeri*−) at each temperature. Hence, the α-diversity indices Chao1-bc (species richness), PD (phylogenetic diversity), and Shannon (equality of abundance) all tended to increase following the experimental manipulations at 28 °C (Figure 6). In contrast, at 20 °C, these indices either decreased slightly in response to pathogen challenge (injury−, *Y. ruckeri*+) or decreased drastically following both injury and pathogen challenge (injury+, *Y. ruckeri*+) reflecting occupancy by *Y. ruckeri*.

### 3.4. Effects of Y. ruckeri on the Members of the Skin Bacterial Flora

As we observed that *Y. ruckeri* infection alters the zebrafish skin bacterial flora, we investigated the relationship between members of the skin bacterial flora and this pathogen. To do so, we collected bacteria from zebrafish skin and determined the direct effects of *Y. ruckeri* on their growth on agar medium at both 20 °C and 28 °C using a cross-streak method. Our results indicate that *Y. ruckeri* inhibits the growth of some bacteria at both 28 °C and 20 °C (Figure S3). However, we did not observe similar inhibitory activity against fish skin bacteria for three other fish pathogens, namely *A. caviae*, *V. ordalii*, and *A. hydrophila*. The results of the growth inhibition test against tens to over a hundred isolated colonies from zebrafish skin are summarized in Table 1. While the growth of only one of 124 bacterial colonies (0.8%) was inhibited by *Y. ruckeri* at 28 °C, seven of 40 (17.5%) were inhibited at 20 °C. These data indicate that *Y. ruckeri* inhibits the growth of some bacteria in the skin microflora of fish, and that the skin bacteria are more susceptible to inhibition by *Y. ruckeri* at 20 °C than at 28 °C. 

### 3.5. Effects of the Disruption of Skin Bacterial Flora on the Colonization of Y. ruckiri on Zebrafish Skin

**Figure 7:**
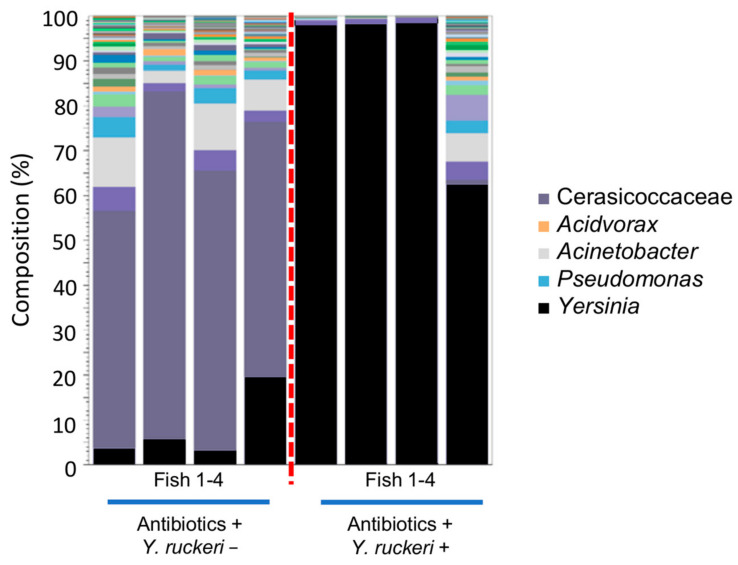
Composition of skin bacterial floras from zebrafish treated with an antibiotic cocktail. The stacked bar chart shows the relative abundance of OTUs (top 50) in skin bacterial floras sampled from antibiotic-treated fish. The samples were grouped by the status of the pathogen challenge (*Y. ruckeri* + or −).

To investigate how the intrinsic skin bacterial flora affects colonization and percutaneous infection by *Y. ruckeri*, we aimed to disrupt the skin bacterial flora using an antibiotic cocktail. The amount of 16S rDNA contained in fish skin samples decreased significantly 24 h after placing the fish in antibiotic cocktail water at 20 °C, and this effect lasted for a week while breeding the fish in sterilized water (Figure S4). We thereby verified that the skin bacterial flora was disrupted by the antibiotic treatment. Zebrafish that had either been treated with antibiotics or left untreated were exposed to *Y. ruckeri* for 6 h at 20 °C. Seven days after this pathogen challenge, the skin of three individuals among the four fish treated with the antibiotics were almost fully occupied by the genus *Yersinia*. Moreover, *Yersinia* spp. became the dominant species on the other individual, making up more than 60% of the bacterial population on its skin (Figure 7). However, none of these four fish died following the pathogen challenge (Figure S5), probably because they were not injured before the challenge. In the absence of pathogen challenge, the genus *Yersinia* was not predominant on the skin of antibiotic-treated fish. 

## 4. Discussion

In this study, we found that pricking zebrafish with a needle did not result in *Y. ruckeri* infection following pathogen challenge. This bacterium had not been reported to be pathogenic toward zebrafish when we started this study. The infection of injured zebrafish by *Y. ruckeri* was caused by cold stress due to a shift in breeding water temperature from the optimum 28 °C to 20 °C. Most fish subjected to these experimental manipulations died and symptoms of ERM were observed in the dead fish, indicating that *Y. ruckeri* can cause the fatal infection of zebrafish. 

We verified the adhesion of this bacterium to the skin of infected fish (Figure 2 and Figure S1), despite low numbers of *Y. ruckeri* in the breeding water (Figure 3). *Y. ruckeri* is known to form biofilms on solid surfaces, and such biofilms are believed to be the source of recurrent infection in aquaculture facilities [12]. The *in vivo* observation of *Y. ruckeri* (Figure 2) suggests that a substantial population of *Y. ruckeri* directly colonized the skin of injured zebrafish at 20 °C. Furthermore, *Y. ruckeri* either occupied zebrafish skin or dominated the skin bacterial flora under infectious conditions (injury+, pathogen challenge+, 20 °C). Disruption of skin bacterial flora by antibiotic treatment also resulted in either the *Y. ruckeri* occupying zebrafish skin or becoming predominant among the fish skin bacterial flora. This result suggests that the intrinsic skin bacterial flora protects zebrafish from pathogen adhesion and colonization. 

As we have demonstrated, *Y. ruckeri* infection of zebrafish requires cold stress, which changes the composition of the skin bacterial flora drastically, including the major genera represented (Figure 4 and Figure 5 and Figure S2, Table S2). This transition may allow the pathogen to dominate the fish skin bacterial flora. The change in bacterial floras on zebrafish was small or ambiguous by pathogen challenge or injury alone, which did not specifically increase *Y. ruckeri* on fish skin (Figure 5A,B). Both injury and pathogen challenge only had slight effects on the skin bacterial flora: population ratios of the genera *Acidovorax* and *Luteolibacter*, whose OTUs were not recognized without injury and pathogen challenge, increased, but the population ratio of the genus *Yersinia* did not increase significantly (Figure 5C). Neither *Acidovorax* nor *Luteolibacter* have been reported to antagonize fish pathogens’ growth as far as we know. However, these bacteria may play a role in repairing damage to the skin bacterial flora by pathogen challenge as a phenomenon of microflora resilience by colonizing a niche on the skin. 

Environmental perturbations often challenge bacterial flora homeostasis. After such perturbations, a resilient bacterial flora will return to its original equilibrium or else dysbacteriosis would result [26]. The resilience of microflora may be suppressed by a shift in temperature to 20 °C. The trends of the α-diversity indices at 20 °C (Figure 6) indicate that cold stress destabilized the skin microflora of zebrafish and enabled the pathogen to affect the fish more easily. Once *Y. ruckeri* colonizes a niche delivered by a change in the microflora, this bacterium may increase in proportion exclusively on the fish skin, resulting in its occupation or domination of the microflora as well as a decrease in α-diversity (Figure 6). The reason for *Y. ruckeri*’s occupation or domination of the skin microflora may be due to its ability to inhibit the growth of some bacteria present on zebrafish skin (Figure S3), especially at 20 °C (Table 1).

The reason for the drastic change in the skin bacterial flora caused by the change in temperature remains unclear. This is probably mainly because the optimum growth temperature varies among bacterial species. Alteration of the host’s immune system by cold stress may also be a factor. Fish are cold-blooded animals, and water temperature is known to affect immune responses, including antibody production [27,28,29]. Zebrafish immune activity could be negatively impacted by lowering the water temperature. For example, skin mucus secreted from the epithelial cells contains biological defense factors such as lectin [1,2,30,31,32,33]. Such immune responses, including the secretion of defense factors, may be less active at 20 °C, leading to changes in the composition of the skin bacteria flora. 

The result of the antibiotic treatment experiment shown in Figure 7 indicates that disruption of the intrinsic skin bacterial flora promotes *Y. ruckeri* adhesion to the skin and made it the predominant species in bacterial flora reconstructed on the fish skin following disruption. Although the fish skin was occupied by *Y. ruckeri* following disruption of the skin bacterial flora with antibiotics (Figure 7), the fish survived (Figure S5). This is likely due to the fact that there was no injury to provide an invasion route for this pathogen through the skin. Invasion into the fish body is necessary for *Y. ruckeri* to cause ERM in zebrafish. However, the abundance of *Yersinia* on the skin did not increase unless the injury was inflicted even at 20 °C (Figure S6), suggesting that the injury not only provided an invasion route for *Y. ruckeri* but also imposed stress on the zebrafish, which together with the temperature shift, allowed this pathogen to dominate the skin bacterial flora.

## 5. Conclusions

Our experimental data indicate that *Y. ruckeri* colonizes niches on the skin surface generated by transient disruption of the skin microflora caused by stress. Moreover, this pathogen can dominate the skin bacterial flora, occupy the surface of fish skin, invade the fish body following injury, and finally cause fatal ERM. We therefore established a percutaneous infection model using zebrafish and *Y. ruckeri*. This model can be used to study the interaction between fish skin bacterial floras and fish pathogens in water, or the relationship between pathogens and the host’s skin immune system.

## Figures and Tables

**Table 1:** Growth inhibition of fish skin bacteria by *Y. ruckeri.*

Origin	Screening Temperature	Number of Acquired Colonies	Test Temperature	Growth Inhibition by *Y. ruckeri*	Ratio (%)
Zebrafish (28 °C)	28 °C	124	28 °C	1	0.8
Zebrafish (28 °C)	20 °C	40	20 °C	7	17.5

## Data Availability

The data presented in this study are openly available in the DDBJ Sequenced Read Archive under the accession number DRX254684-DRX254701.

## Supplementary Materials

The following are available online at https://www.mdpi.com/2079-7737/10/2/166/s1, Figure S1: Detection of *Yersinia ruckeri* on zebrafish skin by PCR, Figure S2: Other angles of 3D-PCoA analysis shown in Figure 4, Figure S3: Growth inhibition assay by fish pathogens against bacterial strains isolated from zebrafish skin, Figure S4: Detection of *Y. ruckeri* on the skin of zebrafish treated with an antibiotic cocktail, Figure S5: The survival rate of fish in infection experiments with antibiotics-treated fish, Figure S6: Composition of skin bacterial floras from zebrafish maintained at 20 ℃ after *Y. ruckeri* challenge, Table S1: Primers used in this study, Table S2: PARMANOVA for β-diversity analysis between the experimental groups, Data S1: observed OTUs at 20 °C and 28 °C, Data S2: alpha diversity rarefaction curves.
