# Supplementary material for: Establishing a Percutaneous Infection Model Using Zebrafish and a Salmon Pathogen
Source: Biology (Basel). 2021 Feb 22;10(2):166. doi: 10.3390/biology10020166 (PMC7926712; doi:10.3390/biology10020166)
Supplement: Supplementary file 1 [file biology-10-00166-s001.zip › biology-1093227-suppl-final/biology-1093227-suppl-final.pdf]

biology

Supplemental Materials

Establishing a Percutaneous Infection Model Using Zebrafish and A Salmon Pathogen

Hajime Nakatani and Katsutoshi Hori

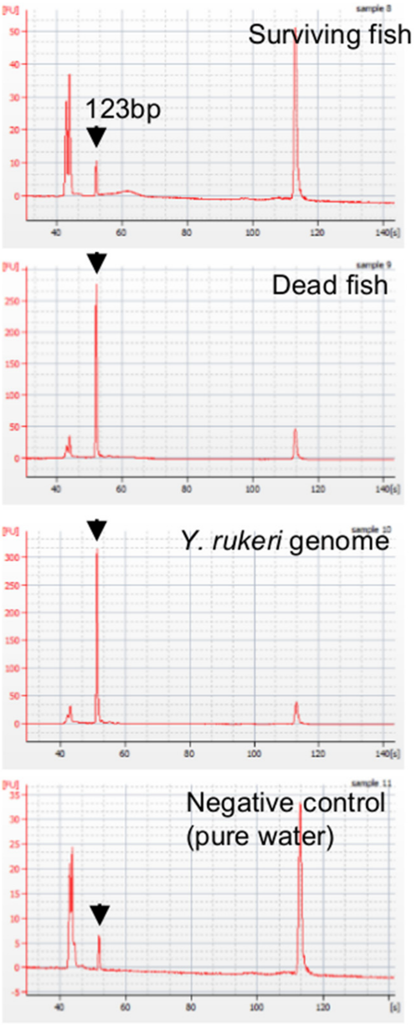

Figure S1. Detection of *Yersinia ruckeri* on zebrafish skin by PCR. The *Y. ruckeri glnA* gene was amplified by 30 cycles of PCR from skin samples of the injured fish surviving and dead fish. The size of amplified DNA was determined by using an Agilent 2100 Bioanalyzer.

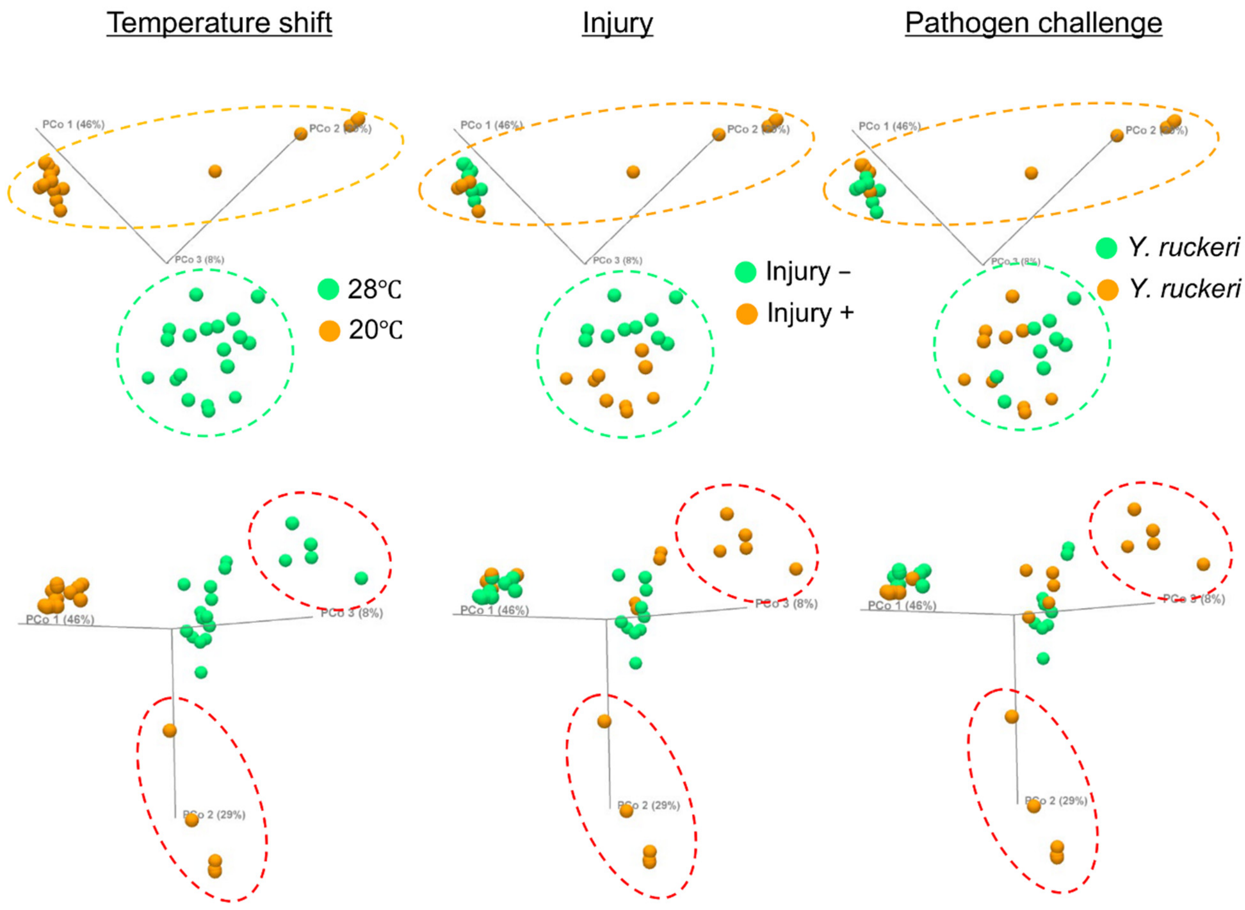

Figure S2. Other angles of 3D-PCoA analysis shown in Figure 4. The β-diversity of fish skin bacterial floras in infection experiments were calculated by using weighted UniFrac distances, and the results are shown in a 3D-PCoA plot. Green dotted circles, orange dotted circles, and red dotted circles represent fish grouped by the test temperature 28℃, the test

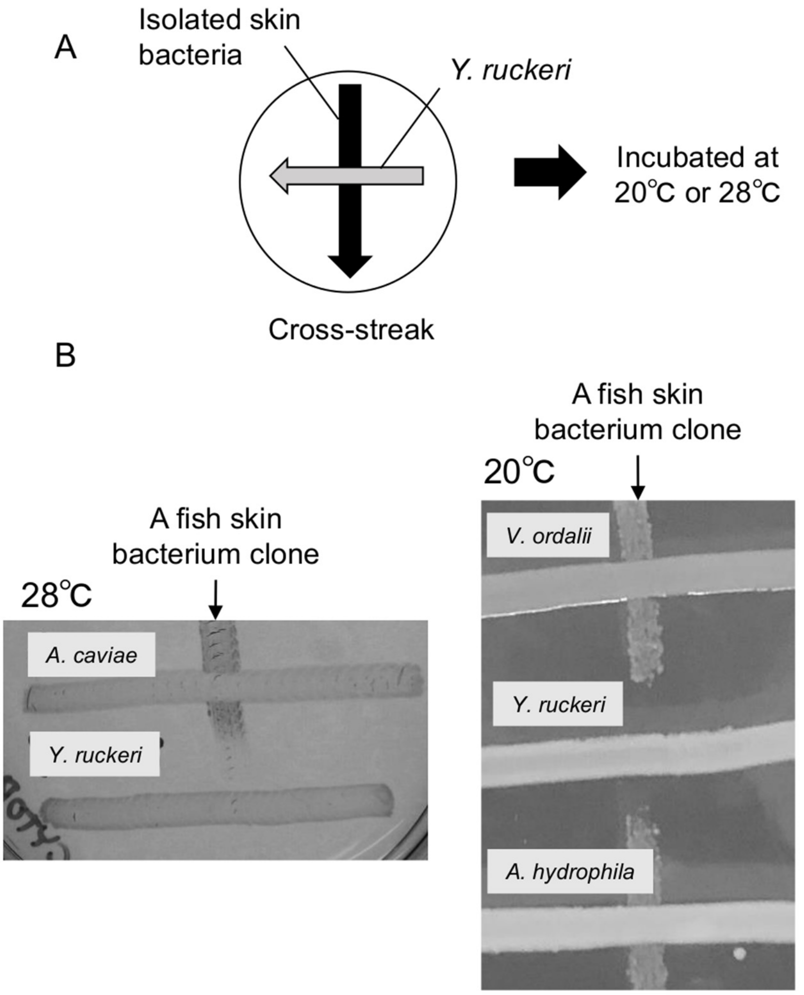

Figure S3. Growth inhibition assay by fish pathogens against bacterial strains isolated from zebrafish skin. (A) Schematic illustrating the cross-streak method for determining antagonistic effects of *Y. ruckeri* towards bacteria isolated from zebrafish skin. (B) Examples of results obtained at 20°C and 28°C. Note that the growth of skin bacteria was interrupted in the vicinity of *Y. ruckeri*.

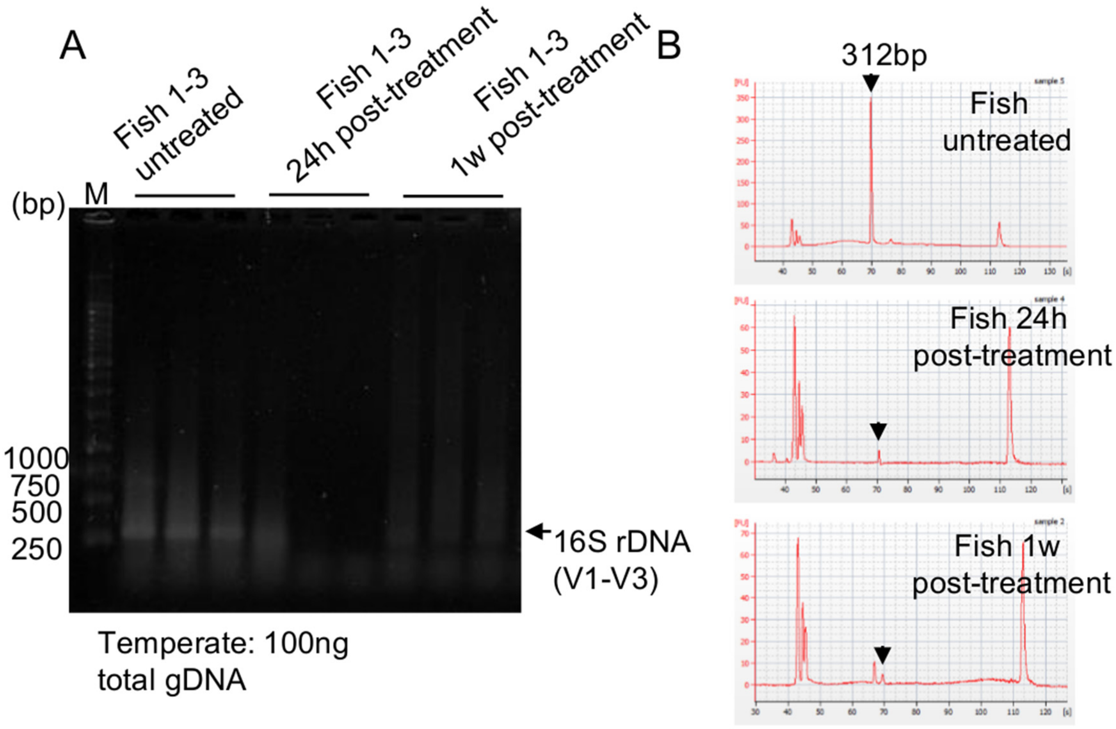

Figure S4. Detection of *Y. ruckeri* on the skin of zebrafish treated with an antibiotic cocktail. The bacterial 16S rDNA V1–V2 region was detected by 30 cycles of PCR before, 24 h after, and 7 d after the treatment. The size of amplified DNA fragments was determined by agarose gel electrophoresis (A) and an Agilent 2100 Bioanalyser (B).

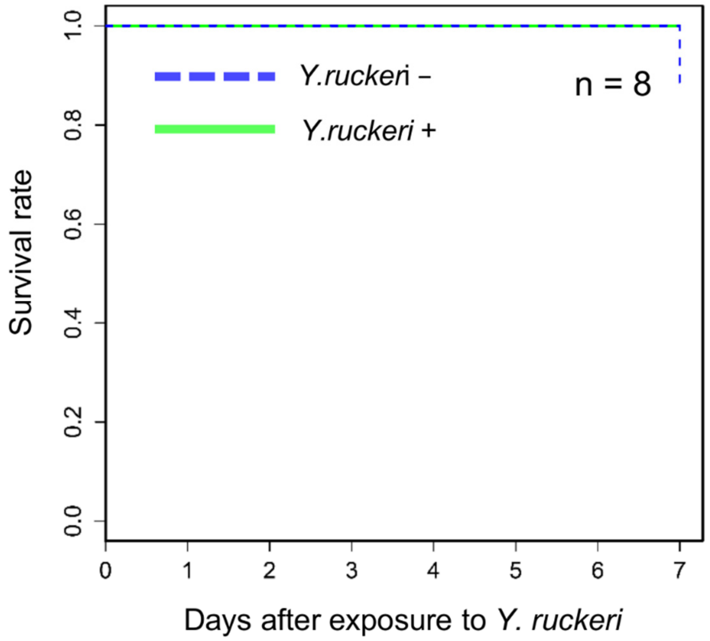

Figure S5. The survival rate of fish in infection experiments with antibiotics-treated fish. Surviving fish was counted every day for 7 d after the challenge of *Y. ruckeri*. The survival rate of the fish that had been subject to pathogen challenge (green line) was compared with the fish that had not been challenged (blue dotted line).

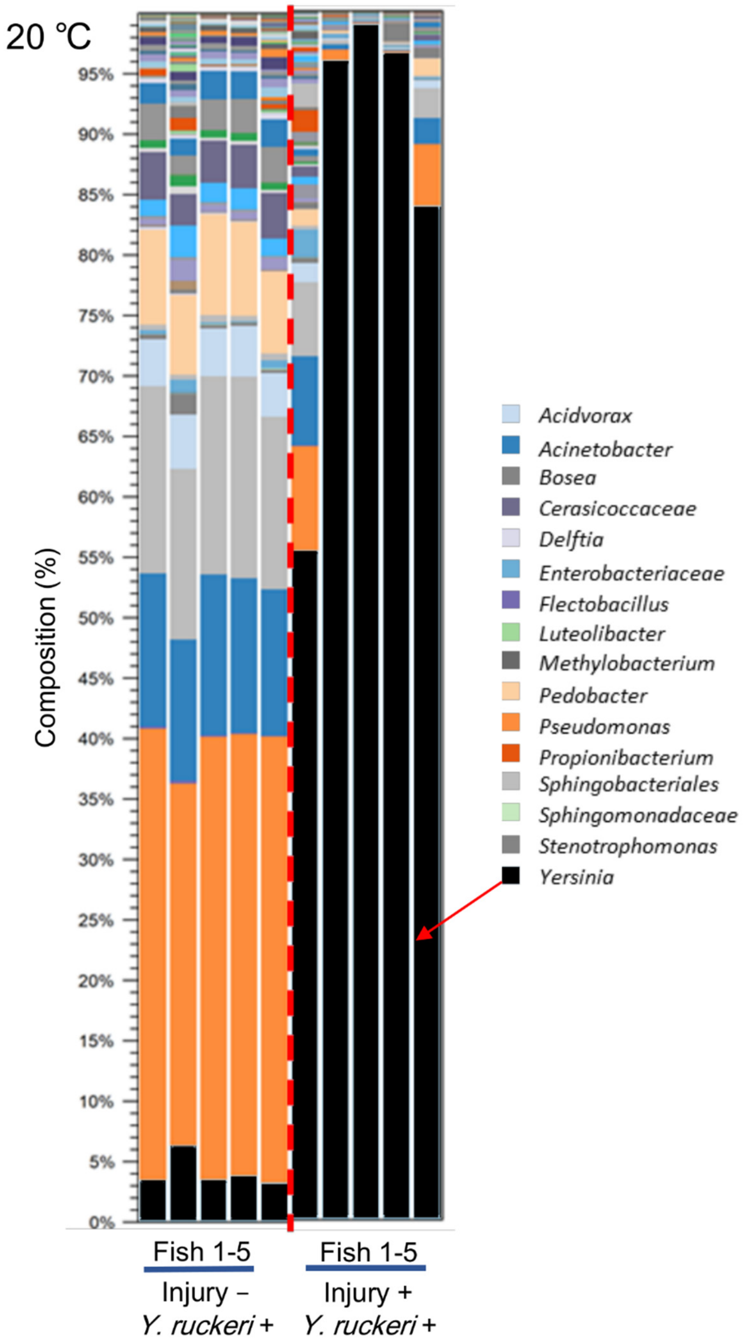

Figure S6. Composition of skin bacterial floras from zebrafish maintained at 20°C after *Y. ruckeri* challenge. The stacked bar chart shows the relative abundance of OTUs (top 50) in skin bacterial flora sampled from uninjured fish (injury –, *Y. ruckeri* +) and from injured fish (injury + *Y. ruckeri* +) 7 d after pathogen challenge.

Table S1. Primers used in this study.

Primer Name	Sequence (5′–3′)	Source or reference
NGS Forward primer	TCGTCGGCAGCGTCAGATGTGTATAAGAGA-CAGGTGYCAGCMGCCGCGGTAAT	Earth Microbiome Project*
NGS Reverse Primer	GTCTCGTGGGCTCCGGAGATGTGTATAAGAGACAGGGAC-TACHVGGGTWTCTAAT	Earth Microbiome Project*
63F	CAGGCCTAACACATGCAAGTC	–
338R	GCTGCCTCCCGTAGGAGT	–
YR glnA F	GTATTGATGATGTACATAAGGAAATGAAACGAGC	Keeling et al.**
YR glnA R	CATTTCCTTATGTACATCATCAATACGGTTG	Keeling et al.**

* http://www.earthmicrobiome.org/emp-standard-protocols/16s/; ** Keeling SE, et al. *J Fish Dis*. 2012;35(2):119-125. doi:10.1111/j.1365-2761.2011.01327.x.

Table S2. PARMANOVA for β-diversity analysis between the experimental groups.

Groups	Pseudo-F statistic	P value (Bonferroni)
20°C vs. 28°C	26.16	0.00001
Injury − vs.Injury + (28℃)	4.13	0.048
Injury − vs. Injury + (20℃)	2.99	0.62
Y. ruckeri − *vs Y. ruckeri* + (28℃)	3.90	0.048
Y. ruckeri − vs *Y. ruckeri* + (20℃)	2.04	0.34
Injury+*Y. ruckeri* vs. others (28℃)	16.27	0.00009
Injury+*Y.ruckeri* vs. others (20℃)	195.45	0.00012
